# Simplifying the design of microstructured optical fibre pressure sensors

**DOI:** 10.1038/s41598-017-03206-w

**Published:** 2017-06-07

**Authors:** Jonas H. Osório, Giancarlo Chesini, Valdir A. Serrão, Marcos A. R. Franco, Cristiano M. B. Cordeiro

**Affiliations:** 1 Instituto de Física ‘Gleb Wataghin’, Universidade Estadual de Campinas, Unicamp, Brazil; 2 Instituto de Estudos Avançados, IEAv, Departamento de Ciência e Tecnologia Aeroespacial, São José dos Campos, Brazil

## Abstract

In this paper, we propose a way to simplify the design of microstructured optical fibres with high sensitivity to applied pressure. The use of a capillary fibre with an embedded core allows the exploration of the pressure-induced material birefringence due to the capillary wall displacements and the photoelastic effect. An analytical description of pressure-induced material birefringence is provided, and fibre modal characteristics are explored through numerical simulations. Moreover, a capillary fibre with an embedded core is fabricated and used to probe pressure variations. Even though the embedded-core fibre has a non-optimized structure, measurements showed a pressure sensitivity of (1.04 ± 0.01) nm/bar, which compares well with more complex, specially designed fibre geometries reported in the literature. These results demonstrate that this geometry enables a novel route towards the simplification of microstructured fibre-based pressure sensors.

## Introduction

The application of fibre optics in sensing applications has been widely studied in recent years. Hydrostatic pressure, temperature, refractive index, strain and curvature are some examples of the parameters that can be monitored by using optical fibres^1^. In this context, numerous technologies – such as Bragg and long-period gratings^2, 3^, fibre tapers^4^, multimode interferometers^5^, rocking filters^6, 7^, photonic-crystal fibres (PCFs)^8, 9^ or combinations thereof – have been used to explore the potential of optical fibres in performing sensing measurements. Advantages of optical fibre-based sensors include high sensitivity, electromagnetic immunity and the possibility of functioning in harsh environments. Moreover, they are usually very compact, lightweight and provide great liberty with respect to choosing a sensor’s characteristics^1^.

Due to their inherent design versatility, microstructured optical fibres are a highly suitable platform for realizing pressure sensors^6–9^. In this approach, they are usually designed in such a manner that the application of hydrostatic pressure generates asymmetric stress distributions within a fibre and, via the photoelastic effect^10^, birefringence variations. Side-hole PCFs^9^ and fibres with sophisticated microstructure geometries (such as the fibre reported by A. Anuszkiewicz *et al*.^8^, which is endowed with a triangular-shaped pattern of air holes), for instance, have already been used to probe pressure variations. The fabrication process of these fibres is, however, complicated and time-consuming and demands much technical effort.

In this paper, we propose the use of a simplified specialty optical fibre structure – the embedded-core capillary fibre – in hydrostatic pressure measurements. The proposed fibre consists of a silica capillary structure with a germanium-doped region (which acts as the fibre core) placed within the capillary wall. Due to the simplicity of the fibre, its fabrication process is straightforward and can be accomplished in a single-step drawing process.

Typical microstructured optical fibres used in pressure sensors are usually endowed with air holes in patterns whose geometric characteristics are chosen so that they can asymmetrically shield the fibre core from the effect of pressure and generate birefringence variations within the core. Here, we avoid the use of a pattern of holes and work with a capillary fibre. The photoelastic effect is explored by simply studying the capillary walls’ displacement due to the application of external pressure and the induced variations in the stress profile.

In the following sections, an investigation of pressure-induced birefringence in capillary fibres is presented. Initially, material birefringence is analytically studied using the Lamé solution^11^ for the stresses within pressurized tubes. In sequence, modal birefringence is studied numerically. Finally, the fabrication of the fibre is described, and experimental pressure-sensing results are reported and compared to simulated data and literature values. The results show that pressure-induced birefringence variations attained in embedded-core capillary fibres are similar to those achieved in sophisticated microstructured fibres. An important step towards the simplification of microstructured fibre-based pressure sensors can, therefore, be identified.

## Results

### Pressure-induced material birefringence in capillary fibres

**Figure 1:**
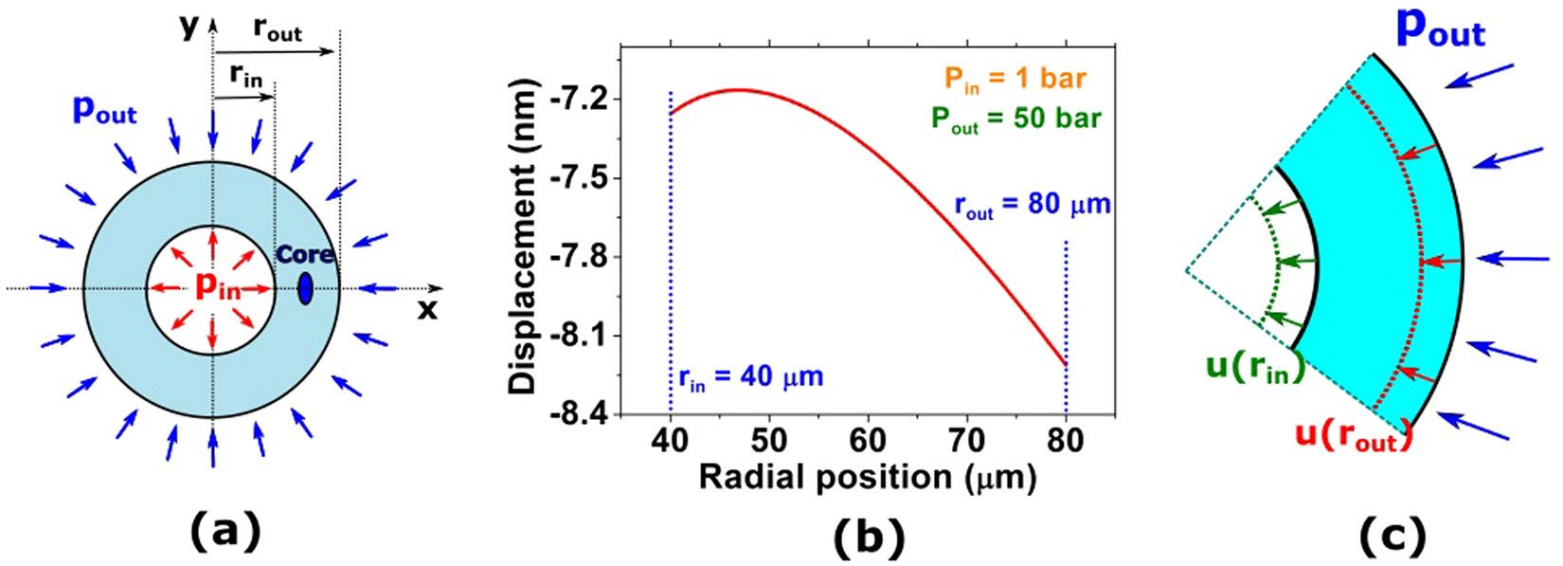
Capillary fibre under pressure. (**a**) Diagram of the pressurized capillary fibre with an embedded core. (**b**) Displacement as a function of the radial position for a capillary fibre with *r*_*in*_ = 40 μm and *r*_*out*_ = 80 μm under an external pressure of 50 bar and internal pressure level of 1 bar. (**c**) Schematic of the observed displacement at the inner and outer radii due to the application of pressure.

The application of hydrostatic pressure to capillary fibres (Fig. 1a) causes displacements on their walls, which implies the induction of stresses into the capillary structure^11^. As material birefringence is a function of the induced stresses, its value is expected to vary if the fibre undergoes pressure. Equation 1 expresses material birefringence, *B*_*mat*_, dependence on the pressure-induced stresses in the horizontal and vertical directions, i.e., *σ*_*x*_ and *σ*_*y*_, respectively^12^. In Equation 1, *C*_*1*_ and *C*_*2*_ are the elasto-optic coefficients; *n*_*x0*_ and *n*_*y0*_ are the material refractive indexes under no stress. For silica, the values are C_1_ = −0.69 × 10^−12^ Pa^−1^ and C_2_ = −4.19 × 10^−12^ Pa^−1^ ^13^.

1 $${B}_{mat}={n}_{x0}-{n}_{y0}+({C}_{2}-{C}_{1})({\sigma }_{x}-{\sigma }_{y})$$

To study the pressure-induced material birefringence profile inside the wall of capillary fibres, one analysed the Lamé solution for the stresses inside thick-walled tubes subjected to hydrostatic pressure^11^. As already referenced, stresses inside the capillary fibre walls arise from the displacement they experience when hydrostatic pressure is applied. The displacement at a radial position *r* inside the capillary wall, *u(r)*, can be found using Equation 2 – where *r*_*in*_ and *r*_*out*_ are the inner and outer radii of the tube, respectively, *υ* and *E* are the Poisson ratio and Young’s modulus of the material the capillary wall is made of (for silica, *υ* = 0.165 and *E* = 72.5 GPa^5, 14, 15^), respectively, and *p*_*in*_ and *p*_*out*_ are the internal and external pressure applied to the fibre, respectively. Due to the problem symmetry, the displacement does not depend on the azimuthal coordinate and, therefore, is a function of the radial position only^11^.

2 $$u(r)=\frac{1}{E}{[1-{(\frac{{r}_{in}}{{r}_{out}})}^{2}]}^{-1}\{(1-\upsilon )[{p}_{in}{(\frac{{r}_{in}}{{r}_{out}})}^{2}-{p}_{out}]r+(1+\upsilon )({p}_{in}-{p}_{out})\frac{{r}_{in}^{2}}{r}\}$$

Figure 1b shows the displacement profile along the wall of a silica capillary (with the *r*_*in*_ */r*_*out*_ ratio being equal to 0.5) when it is subjected to an external pressure level of 50 bar (5 MPa) and has an internal pressure level of 1 bar (0.1 MPa). In this simulation, *r*_*in*_ is considered to be 40 μm. One can see that for the case shown in Fig. 1b, the displacement at the inner radius position is, in modulus, lower than the one observed at the outer radius position, implying a decrease in wall thickness. This situation is schematized in Fig. 1c, in which the inner and outer wall displacements are represented.

**Figure 2:**
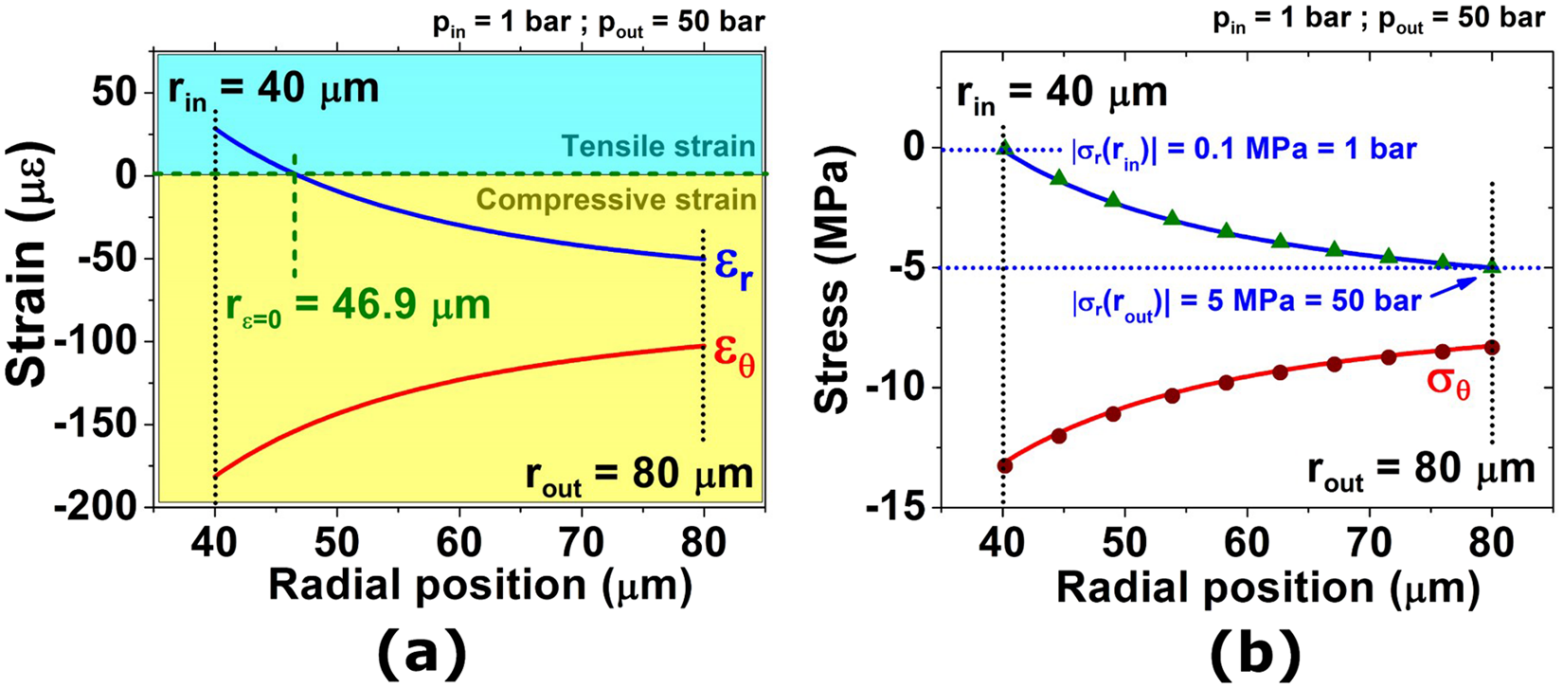
Strain and stresses within capillary fibre wall. (**a**) Strain and (**b**) stress as a function of the radial position for a capillary fibre with *r*_*in*_ = 40 μm and *r*_*out*_ = 80 μm under an external pressure of 50 bar and inner pressure level of 1 bar. Green triangles and dark red circles are the results obtained from the COMSOL^®^ numerical model.

Displacements of the mass elements inside the tube wall allow for defining strains into the fibre structure. According to the Lamé description, the strain along the radial direction is obtained by solving ${\varepsilon }_{r}(r)=\frac{du(r)}{dr}$, whereas the strain along the azimuthal direction is obtained by solving ${\varepsilon }_{\theta }(r)=\frac{u(r)}{r}$, where *u*(*r*) is the displacement shown in Equation 2 ^11^. Considering a situation in which a capillary with inner and outer radii of 40 μm and 80 μm, respectively, is subjected to an external pressure of 50 bar, the strain results are as shown in Fig. 2a. By observing the behaviour of the strain along the radial direction, it can be observed that it can be either tensile (${\varepsilon }_{r}(r) > 0$) or compressive (${\varepsilon }_{r}(r) < 0$). There is a point of zero radial strain at the radial position ${r}_{{\varepsilon }_{r}=0}={r}_{in}\sqrt{\frac{(1+\upsilon )({p}_{out}-{p}_{in})}{(1-\upsilon )}{[{p}_{out}-{p}_{in}{(\frac{{r}_{out}}{{r}_{in}})}^{2}]}^{-1}}$, which, for the situation shown in Fig. 2a (*r*_*in*_ = 40 μm, *r*_*out*_ = 80 μm, *p*_*in*_ = 1 bar, *p*_*out*_ = 50 bar and *υ* = 0.165), is calculated to be 46.9 μm. Moreover, one can observe that the azimuthal strain is always compressive.

The Lamé description also allows the determination of the radial (*σ*_*r*_) and hoop (*σ*_θ_) stresses, which are induced within the tube wall due to the application of hydrostatic pressure. The results are expressed by Equations 3 and 4, where *r*_*in*_ and *r*_*out*_ represent the inner and outer radii of the tube, respectively, and *p*_*in*_ and *p*_*out*_ represent the internal and external pressure, respectively. Again, due to the problem symmetry, the stresses do not vary azimuthally and are a function of the radial position only^11^.

3 $${\sigma }_{r}(r)={[1-{(\frac{{r}_{in}}{{r}_{out}})}^{2}]}^{-1}\,[{p}_{in}{(\frac{{r}_{in}}{{r}_{out}})}^{2}-{p}_{out}-({p}_{in}-{p}_{out}){(\frac{{r}_{in}}{r})}^{2}]$$

4 $${\sigma }_{\theta }(r)={[1-{(\frac{{r}_{in}}{{r}_{out}})}^{2}]}^{-1}\,[{p}_{in}{(\frac{{r}_{in}}{{r}_{out}})}^{2}-{p}_{out}+({p}_{in}-{p}_{out}){(\frac{{r}_{in}}{r})}^{2}]$$

Figure 2b shows the radial and hoop stress behaviours along the tube wall (between r_in_ = 40 μm and r_out_ = 80 μm) for the situation in which the tube wall is subjected to an external pressure of 50 bar and an internal pressure of 1 bar (according to Equations 3 and 4). Note that the absolute value of the radial stress at the outer radius assumes the external pressure value (*p*_*out*_ = 50 bar = 5 MPa), whereas that at the inner radius assumes the internal pressure value (*p*_*in*_ = 1 bar = 0.1 MPa), which are the boundary conditions of the problem. Furthermore, numerical data attained from a finite-element-based model built using the COMSOL^®^ software are also shown in Fig. 2b (green triangles and dark red circles). It is seen that the analytical and numerical results are very similar.

Stresses written in polar coordinates (*σ*_*r*_ and *σ*_θ_) can be readily expressed in rectangular coordinates (*σ*_*x*_ and *σ*_*y*_) by employing the transformations presented in Equation 5 ^11^. To attain *σ*_*x*_ and *σ*_*y*_ along the horizontal axis, one sets θ = 0 and, therefore, concludes that *σ*_*r*_ = *σ*_*x*_ and *σ*_θ_ = *σ*_*y*_.

5 $${\sigma }_{x}={\sigma }_{r}\,{\cos }^{2}\,\theta +{\sigma }_{\theta }\,{\sin }^{2}\,\theta ;\,{\sigma }_{y}={\sigma }_{r}\,{\sin }^{2}\,\theta +{\sigma }_{\theta }\,{\cos }^{2}\,\theta $$

By identifying that along the horizontal axis, *σ*_*x*_ can be expressed by Equation 3 and *σ*_*y*_ by Equation 4, and by substituting these results into Equation 1, one can obtain Equation 6, which expresses the behaviour of the material birefringence at a position *x* along the horizontal axis inside the capillary wall. Moreover, for obtaining Equation 6, as shown below, it is assumed that *n*_*x*0_ and *n*_*y*0_ are identical (no birefringence under no stress). Additionally, one defines gauge pressure as *p*_*gauge*_ ≡ *p*_*out*_ − *p*_*in*_.

6 $${B}_{mat}(x)=2({C}_{2}-{C}_{1}){p}_{gauge}{[1-{(\frac{{r}_{in}}{{r}_{out}})}^{2}]}^{-1}\frac{{r}_{in}^{2}}{{x}^{2}}$$

**Figure 3:**
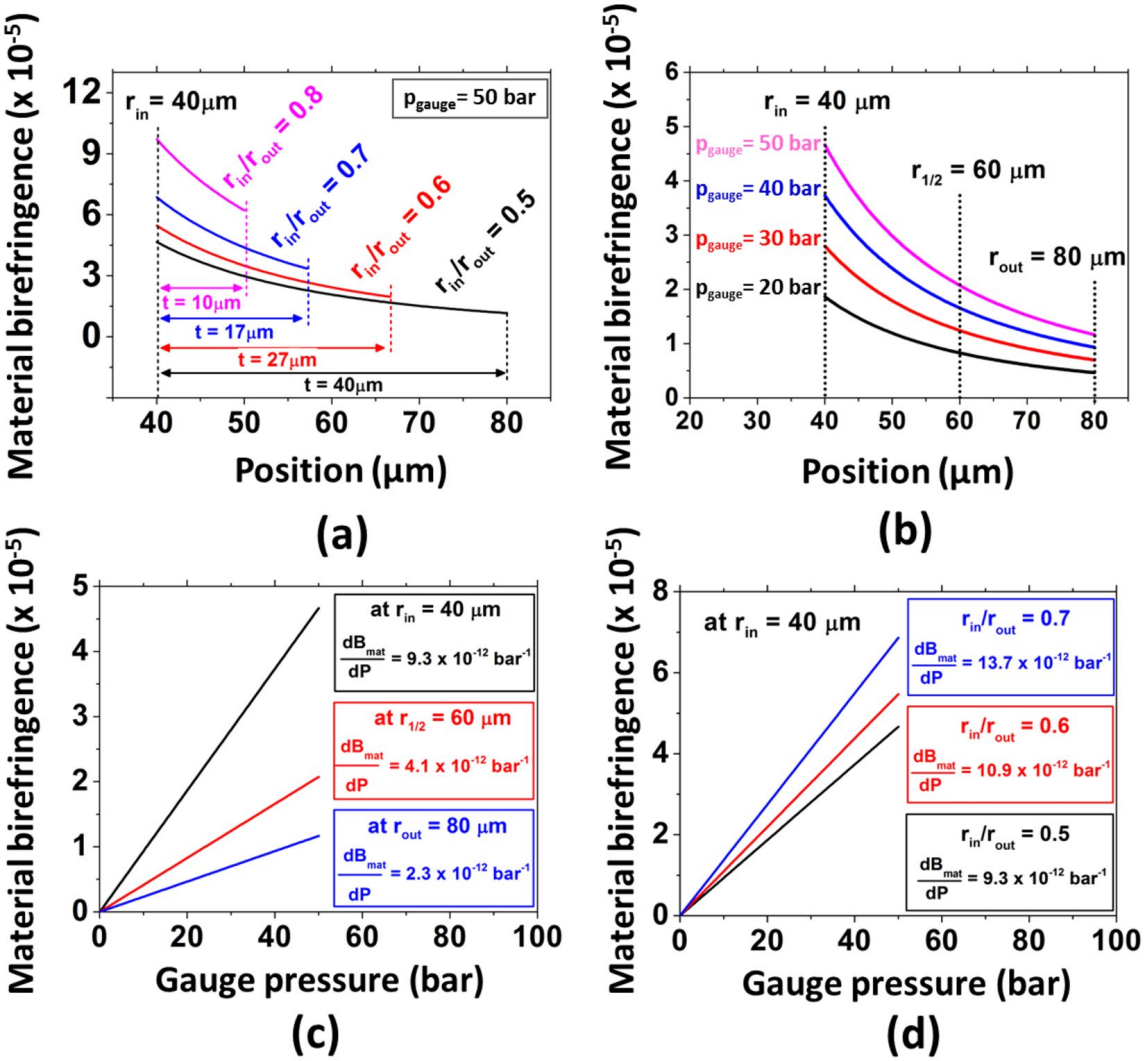
Study of pressure-induced material birefringence. (**a**) Material birefringence along the horizontal axis for capillaries with different *r*_*in*_ */r*_*out*_ ratios and (**b**) for a capillary with *r*_*in*_ = 40 μm and *r*_*out*_ = 80 μm under increasing gauge pressure values. (**c**) Material birefringence as a function of gauge pressure at the positions *r*_*in*_ = 40 μm, *r*_*1/2*_ = 60 μm, *r*_*out*_ = 80 μm and (**d**) for a capillary with *r*_*in*_ = 40 μm and a *r*_*in*_ */r*_*out*_ ratio equal to 0.5, 0.6 and 0.7.

If the absolute value of material birefringence is plotted as a function of the position on the horizontal axis for capillaries with different *r*_*in*_ */r*_*out*_ ratio values (but with the same inner radius value, i.e., 40 μm) for *p*_*gauge*_ = 50 bar, one obtains the results shown in Fig. 3a. The results show that when external pressure acts on the fibre, induced material birefringence values are higher for capillary fibres with thinner wall thicknesses. Furthermore, greater material birefringence values are attained for positions closer to the inner radius.

Figure 3b presents material birefringence modulus as a function of the position along the horizontal axis for a capillary with inner and outer core dimensions of 40 μm and 80 μm, respectively, subjected to four different pressure levels (20, 30, 40 and 50 bar). One can realize that although material birefringence increases for all positions inside the capillary wall, the increment occurs at different rates – they are higher closer to the internal side of the capillary and lower towards the external side.

Different slopes of the material birefringence variation due to external pressure application are clearly observed in Fig. 3c, where the material birefringence of a capillary fibre is plotted for the inner radius (*r*_*in*_ = 40 μm), outer radius (*r*_*out*_ = 80 μm), and middle point of the capillary wall (*r*_*1/2*_ = 60 μm). Slopes varied from 9.3 × 10^−12^ bar^−1^ to 2.3 × 10^−12^ bar^−1^ along the capillary fibre wall. Additionally, as shown in Fig. 3d, material birefringence is plotted as a function of the applied external pressure for capillaries with *r*_*in*_ = 40 μm but different *r*_*in*_ */r*_*out*_ ratios. Based on this graph, one can see that *dB*_*mat*_ */dP* is higher for higher *r*_*in*_ */r*_*out*_ ratios. Therefore, it is observed that material birefringence shows a stronger dependence on pressure variations for positions closer to the inner radius and for capillaries with thinner walls.

### Modal birefringence dependence on applied pressure

To explore the birefringence dependence on the applied pressure in practical applications, one studied a configuration in which a high refractive index core is embedded into the capillary wall, as shown in Fig. 1a. The core consists of a germanium-doped region and has an elliptical shape. Although the material birefringence has been analytically studied above, it is necessary to investigate the modal birefringence behaviour. To do this, a capillary fibre with an embedded-core structure was simulated in COMSOL^®^, and its modal birefringence dependency on applied pressure was obtained.

**Figure 4:**
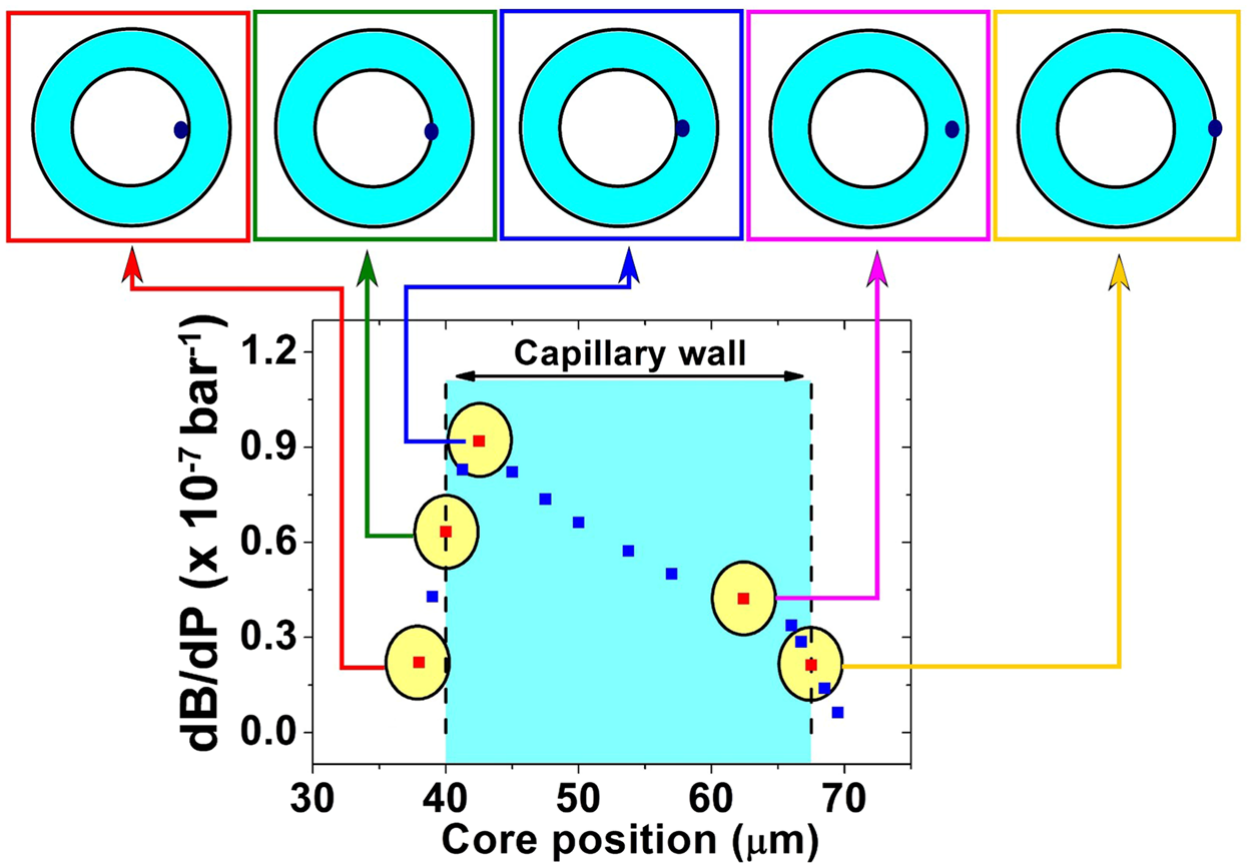
Modal birefringence simulation results. Modal birefringence derivative as a function of the core position in the capillary fibre wall. Blue region represents the capillary wall region, whereas yellow ellipses represent the core area. Insets illustrate the core location within the capillary fibre.

In the simulations, a capillary fibre with inner and external radii of 40 μm and 67.5 μm, respectively, was used. Moreover, the core was considered to be an ellipse with dimensions of 5.7 μm and 11.4 μm. The effective refractive indexes of the *x*- and *y*-polarized core modes were obtained for different pressure conditions, and the birefringence derivative as a function of pressure, *dB*_*modal*_ */dP*, was numerically modelled. The calculation of *dB*_*modal*_ */dP* was performed for several core positions inside the capillary wall, as seen in Fig. 4. The blue region represents the capillary wall, and the yellow ellipses represent the core area. Moreover, the insets in Fig. 4 illustrate the core location (dark blue ellipses) within the capillary structure for selected points from the Fig. 4 plot.

As shown in Fig. 4, one can see that when the entire core is located in the capillary wall, the *dB*_*modal*_ */dP* behaviour is analogous to that of the material birefringence case – it increases towards the inner capillary wall. As the core approaches the inner or outer wall of the capillary, a section of its area can be outside the capillary structure (see Fig. 4 insets), and decreasing *dB*_*modal*_ */dP* values are observed. It is recognized that to obtain a maximized birefringence dependence on pressure variations, it is crucial to completely embed the core area within the capillary structure. As expected from the analytical description, the highest *dB*_*modal*_ */dP* values are found for core positions closer to the inner wall.

### Pressure-sensing measurements

**Figure 5:**
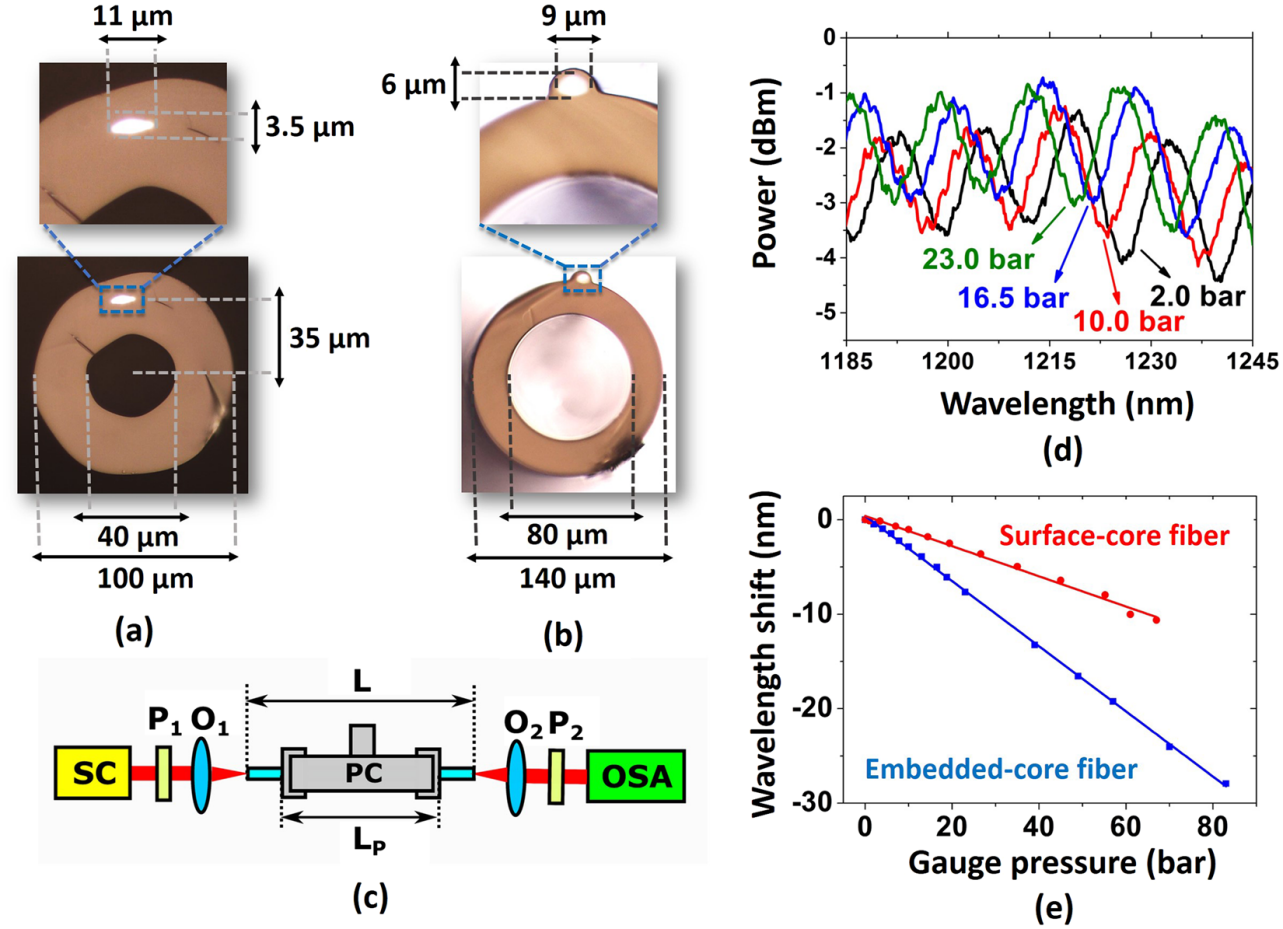
Embedded-core fibres, surface-core fibres and pressure-sensing results. (**a**) Embedded-core fibre and enlargement of the core region. (**b**) Surface-core fibre and enlargement of the core region. (**c**) Experimental setup for pressure measurements. SC: supercontinuum source; P_1_ and P_2_: polarizers; O_1_ and O_2_: objective lenses; OSA: optical spectrum analyser; PC: pressure chamber; L: fibre length; L_P_: pressurized fibre length. (**d**) Spectral response of embedded-core fibre for different pressurization conditions. (**e**) Wavelength shift versus applied pressure for surface-core and embedded-core fibres.

To provide an experimental realization of the proposed fibre structure, a fibre in which the core was embedded into the capillary structure was fabricated. This structure was named an *embedded-core fibre*; Fig. 5a shows its cross-section. The capillary diameters are 40 μm and 100 μm, the distance between the centre of the fibre and the core position is 35 μm, and the core dimensions are 11 μm and 3.5 μm. Additionally, aiming to have a second fibre for comparison, the fabrication of a fibre in which the core was placed on the fibre’s outer surface was performed; this structure was named a *surface-core fibre* ^16^. In this structure, the capillary inner and outer diameters are 80 μm and 140 μm, respectively, and the core dimensions are 6 μm and 9 μm (Fig. 5b). To fabricate the fibres, macroscopic preforms were prepared by merging a germanium-doped silica rod with a silica tube, and the resulting structure was drawn using a tower facility^16^. Both fibres were fabricated at Unicamp.

The polarimetric wavelength scanning method^17^ was used to characterize the sensitivity of the fibres to external pressure variations. In this method, a broadband optical source (supercontinuum from a photonic-crystal fibre – SC) was used to launch light into the optical fibre and a first polarizer (P_1_) was employed for exciting the two orthogonal modes of the birefringent fibre. A second polarizer (P_2_) was used to allow recombination and interference between the orthogonal modes that travelled along the fibre, and the transmitted signal was measured using an optical spectrum analyser (OSA). Moreover, a pressure chamber (PC) was used to subject the fibre to different pressure conditions. Figure 5c presents a schematic of this experimental setup.

The interference spectrum measured using the polarimetric wavelength scanning method is characterized by spectral fringes whose spectral positions are dependent on the pressure applied to the fibre (as embedded-core fibre birefringence is altered when pressure is applied). Thus, one defines a sensitivity coefficient, *C*_*S*_ ≡ *dλ*_*IF*_ */dP*, which accounts for the spectral displacement of the interferometric fringes (IF) due to pressure variations. The *C*_*S*_ value can also be expressed as a function of the wavelength, λ, fibre group birefringence, *G*, and phase birefringence derivative with respect to pressure, *∂B*_*modal*_ */∂P*, as presented in Equation 7 ^9^. By following the spectral positions of the interferometric fringes as the pressure applied to the fibre is varied, the *C*_*S*_ value can be determined.

7 $${C}_{S}\equiv \frac{d{\lambda }_{IF}}{dP}=\frac{\lambda }{G}\frac{\partial {B}_{modal}}{\partial P}$$

Figure 5d shows the spectral response of the embedded-core fibre when it is subjected to pressure variations. One can see that the fringes blueshift as the pressure is increased. In Fig. 5e, blue squares represent the wavelength shift due to pressure application to the embedded-core fibre – fibre length (36.0 ± 0.1) cm and pressurized fibre length (12.0 ± 0.1) cm. Red circles in turn are the pressure-sensing results for the surface-core fibre – fibre length (32.0 ± 0.1) cm and pressurized fibre length (12.0 ± 0.1) cm.

By accounting the wavelength shift as a function of the applied pressure one could obtain, after performing an appropriate correction to the fibre pressurized lengths^9, 18^, pressure sensitivities of (1.04 ± 0.01) nm/bar and (0.43 ± 0.01) nm/bar for the embedded-core and surface-core fibre, respectively. It is seen that the embedded-core fibre sensitivity is 2.4 times that of the surface-core fibre. This result corroborates the simulation results: a maximized sensitivity could be obtained for a fibre with the core placed within the capillary fibre structure.

Regarding the resolution of the sensors, one can observe that the resolution is dependent on the widths of the spectral dips, whose shifts are followed as pressure is applied. In the measurement presented in Fig. 5d, the dip width at half maximum can be estimated as Δλ ≈ 6 nm. Assuming that one can resolve two spectral dips if they are at least Δλ/50 (≈0.1 nm in this case) apart from each other, the system resolution limit can be estimated to be 0.3 bar. As in the polarimetric scanning method, a longer fibre spectrum will have dips with decreased widths; the system’s resolution can be further improved if longer fibres are employed. It is worth observing, however, that the increase in the number of dips causes them to be spectrally closer, which can reduce the dynamic range of measurements. Additionally, increasing the sensor’s length can limit its applicability.

To determine the dynamic range of the embedded-core fibre sensor, one should analyse the spectral separation of the dips (free spectral range – FSR). This analysis is necessary because, in a practical measurement, if a dip reaches the spectral position of a neighbouring one, misinterpretation of the sensor reading can occur. In the measurement presented in Fig. 5d, the spectral range can be estimated as FSR = 14.2 nm. In terms of pressure, this result means that the sensor’s dynamic range is on the order of 40 bar. In this context, it should be emphasized that although one estimates the system’s dynamic range to be 40 bar for practical applications, data up to 80 bar could be measured because, in our experiments, one has carefully followed the spectral positions of the dips as a function of pressure. Additionally, it is worth noting that the dynamic range of measurements, similar to the sensor’s resolution, can be tuned by adequately choosing the fibre length. If, for example, in a specific application the sensing fibre length was 2 cm, its dynamic range would be on the order of 300 bar, making the sensor suitable, for example, for petroleum exploration. The resolution limit, however, would be approximately 5 bar.

Numerical simulations were also performed to provide a comparison with the experimental results. Simulated sensitivity values for the embedded-core and surface-core fibres were obtained using COMSOL^®^ mechanical and optical analyses by calculating the fibre group birefringence and *∂B*_*modal*_ */∂P* using the effective refractive index values. To obtain the most verisimilar analysis possible, one created realistic models based on fibre microscopy images. Simulated sensitivity results were 0.89 nm/bar and 0.50 nm/bar for the embedded-core fibre and surface-core fibre, respectively. A good agreement can be observed between the simulated and experimental values.

As the proposed fibres have a germanium-doped region that acts as the fibre core, temperature sensitivity can also be expected. This result arises from the fact that undoped and doped silica regions present different thermal expansion coefficients and, therefore, temperature variations can affect fibre birefringence^19^. To measure the temperature sensitivity, the fibres were subjected to temperature variations, and their spectral responses were measured by employing the polarimetric wavelength scanning method. The measurements allowed determination of the following values for the birefringence derivative with respect to temperature: *∂B*_*modal*_ */∂T* = (2.8 ± 0.1) × 10^−7^ °C^−1^ for the embedded-core fibre and *∂B*_*modal*_ */∂T* = (4.0 ± 0.4) × 10^−7^ °C^−1^ for the surface-core fibre. These values are similar to the ones reported for commercial polarization-maintaining fibres – 4.0 × 10^−7^ °C^−1^ for the PANDA fibre and 3.6 × 10^−7^ °C^−1^ for the Bow-tie fibre^20^. They are, however, higher than the values attained for specially designed microstructured fibres incorporating a germanium-doped core employed in pressure-sensing measurements (1.7 × 10^−7^ °C^−8^)^21^ and for all-silica photonic-crystal fibres (1.1 × 10^−9^ °C^−1^)^19^.

It is worth observing that if a practical application is targeted, a temperature compensation system would be desirable. This could be done, for instance, by performing the fabrication of an embedded-core fibre with two cores (placed at different radial positions within the capillary wall) and by imprinting a Bragg grating in each one of them. As the core of the embedded-core fibre is obtained from standard optical fibre preforms, a Bragg grating temperature sensitivity on the order of 10 pm/°C could be expected for both cores as in standard Bragg grating sensors^2^. Numerical simulations show that by adequately choosing the cores’ positions within the capillary wall, the pressure sensitivities for Bragg gratings inscribed in the cores could differ by a factor of 2. By taking into account the Bragg grating temperature and pressure sensitivities for both cores, the pressure measurement from the polarimetric measurement could be temperature-compensated.

Additionally, an off-centre core position makes it necessary to conduct further studies on splicing procedures. Moreover, if a practical application is to be targeted, additional studies on sensor packaging would be necessary. A possible approach could be inserting the fibre into a metallic tube with transversal holes on its side. This would protect the fibre and allow the pressure from the external environment to act on the fibre.

## Discussion

By observing the embedded-core fibre sensitivity, it can be seen that this fibre structure is an interesting platform for the performance of pressure-sensing measurements. The experimentally measured sensitivity – (1.04 ± 0.01) nm/bar – is found to be higher than that of other fibre sensors that also focus on polarimetric measurements. For instance, H. Y. Fu *et al*. reported 0.342 nm/bar for a commercial all-silica photonic-crystal fibre^21^, and T. Martynkien *et al*. measured 0.30 nm/bar and 0.52 nm/bar for specially designed microstructured fibres^21, 22^.

Additionally, the birefringence derivative with respect to pressure (*∂B/∂P*) can be estimated to be (2.33 ± 0.02) × 10^−7^ bar^−1^ using Equation 7 for the embedded-core fibre. This value is on the same order of magnitude as those achieved for sophisticated microstructured fibres, such as the photonic-crystal fibre reported by G. Statkiewicz-Barabach *et al*. (*∂B/∂P* = 2.52 × 10^−7^ bar^−1^)^6^ and the specially designed fibre endowed with a triangular pattern of air holes described by A. Anuskiewicz *et al*. (*∂B/∂P* = 8.89 × 10^−7^ bar^−1^)^7^, whose designs were optimized for pressure sensing. The fabrication process of these fibres, however, is much more complex than the one used for obtaining the embedded-core fibres, which requires only a relatively simple fibre drawing process.

Furthermore, one can visualize that there is still room for enhancing the pressure sensitivity of this non-optimized fibre reported herein. Analytical simulations of an embedded-core capillary fibre with, respectively, 70 µm and 100 µm inner and outer diameters and with the core placed 42.5 µm away from the fibre centre (at the middle point of the capillary wall) – all very realistic geometric parameters – were realized. The obtained material birefringence derivative with respect to pressure was 3.4 times the value expected for an embedded-core fibre with the same dimensions as the one we fabricated.

Additional numerical simulations showed that the core asymmetry has little influence on the *dB*_*modal*_ */dP* values. The sensitivity coefficient (*C*_*S*_), however, can be boosted for fibres with more symmetric cores because the guided mode will present lower group birefringence levels. The *C*_*S*_ value for a core with dimensions of 4 µm × 5 µm, for example, is simulated to be approximately 6 times higher than the *C*_*S*_ value for a core with dimensions of 4 µm × 12 µm (these data were found for an embedded-core fibre with an inner radius of 40 µm and outer radius of 67.5 µm, with the core placed at the middle point within the capillary wall).

In conclusion, one can emphasize that in this paper, we provided a new route for the simplification of microstructured optical fibre sensors by exploring the photoelastic effect in capillary fibres. Initially, an analytical description of the pressure-induced material birefringence within a capillary structure was explored. The results demonstrated that an enhanced material birefringence dependence on pressure could be attained for thinner capillaries and at positions closer to their inner walls.

To explore the fibre modal characteristics, numerical simulations were performed, and the results confirmed the analytical simulation predictions. Additionally, it could be noted that the birefringence dependence on pressure variations drops for situations in which part of the core region area is outside the capillary.

Finally, we reported the fabrication of an embedded-core fibre and its use in pressure-monitoring experiments. Measurements revealed a sensitivity of (1.04 ± 0.01) nm/bar and allowed the estimation the birefringence derivative with respect to pressure to be (2.33 ± 0.02) × 10^−7^ bar^−1^. Both the values are very similar to the ones reported for more complicated, specially designed fibre structures. We can visualize, therefore, embedded-core fibres as a very interesting platform with the potential to considerably simplify microstructured optical fibre hydrostatic pressure sensors.

## Methods

### Correction of sensitivity values regarding the pressurized fibre lengths

*C*_*S*_ values denote a situation in which the whole fibre length is pressurized. Because in the experiments only a fraction of the fibre is pressurized (pressurized length, *L*_*P*_), a correction to the experimentally measured sensitivities must be made. It is performed by simply multiplying the measured sensitivities by a factor *L*_*P*_ */L* (where *L* is the fibre length)^9, 18^.

### Embedded-core fibre fabrication procedure

The embedded-core-fibre fabrication process is very similar to that of the surface-core fibre, which was recently reported by the authors^16^. The only difference is that in the embedded-core-fibre fabrication method, an additional jacketing procedure is performed.

Initially, a surface-core preform is prepared by merging a germanium-doped silica rod with the external surface of a silica tube^16^. In sequence, the surface-core preform is inserted into another silica tube, which acts as a jacket. Therefore, the core region is located in between the inner tube and the jacketing one. To obtain the fibre whose cross-section is shown in Fig. 5a, one employed an inner tube with dimensions of 9.5 mm × 11.5 mm and an outer tube with dimensions of 18 mm × 20 mm.

During fibre drawing, a vacuum is applied in the space between the tubes, allowing the tubes to merge. In the merging process, the core is compressed and acquires an elliptical shape. The choice of the germanium-doped rod and tube dimensions for preparing the preform allows planning regarding the core size and its position within the capillary wall.
